# Editor's response

**DOI:** 10.4103/0019-5278.40821

**Published:** 2008-04

**Authors:** G.K. Kulkarni

**Affiliations:** Editor, IJOEM. E-mail: editor@ijoem.com

Dear Sir,

Thank you for your mail. Let me assure you that International Journal of Occupational and Environmental Health (IJOEM) is not silent on the issue. We are aware of the letter published in the said journal by Dr. T.K. Joshi from India. Dr. Joshi has misrepresented the issues and used false premises to defame the IAOH. We have our views on the bonafides and personal agenda of Dr. Joshi, which we are tackling separately.

Dr. Joshi is a member of the IAOH and was the Chair of scientific committee of the 51^st^ National Conference of IAOH in Delhi prior to the IAOH's formal stand on the issue. Based on scientific data, the Central Council of the association took a stand in the year 2003 in favour of Banning Asbestos. The association has not taken any financial support from any industry connected with asbestos after the decision. IJOEM has also supported the Ban Asbestos stand in the Editorial written by me in the issue of Vol. 5, No. 1, January-March 2001. Dr. T.K. Joshi has himself written an editorial in IJOEM Vol. 6, No. 3, July-September 2002 on “Precautionary principle and need to ban all forms of Asbestos use in India”. Now, how can he malign the IAOH and its commitment to advancing the ethical practice of occupational health in India?

In the light of the above, it is indeed surprising that Dr. Joshi has shot of a volley of blatantly misleading information. In the same note, he mentions about no one protecting his job. We wonder if all this is about personal glory or a genuine concern for the health of workers? I leave this for the readers to judge in the light of our response backed by evidence of Dr. Joshi utilizing IAOH's support in his crusade against asbestosis. It may be pertinent to mention here that Dr. Joshi has continued to be associated with us and has accepted awards and invitations, in spite of holding such negative opinions about the association. We wonder what his ulterior motives are. We therefore feel that the views expressed by Dr, Joshi about the IAOH should be taken in the proper perspective and with a pinch of salt. To us in the IAOH, this is a case of a crusade going horribly wrong tinged, as it is with an ulterior motive of one individual projecting himself as a martyr at the altar of occupational health in India. It may win him “brownie points” and in his mind help in holding on to his chair at this institution with the so-called help and support of people outside the country, but it has portrayed Dr. Joshi's institution, the IAOH and indeed the government in poor light - a stand unacceptable to the members of the IAOH.

With respect to the other issues raised by you regarding the functioning of NIOH and the Government of India, they do not fall in the ambit of the IAOH's mandate and we would advise you to correspond with the concerned authorities and get their views. You may also like to take up the issue with the government and try to scientifically convince them rather that raking up such issues in scientific publications and politicizing the scientific community.

